# Cardioprotective Potential of Berries of *Schisandra chinensis* Turcz. (Baill.), Their Components and Food Products

**DOI:** 10.3390/nu15030592

**Published:** 2023-01-23

**Authors:** Beata Olas

**Affiliations:** Department of General Biochemistry, Faculty of Biology and Environmental Protection, University of Lodz, Pomorska 141/3, 90-236 Lodz, Poland; beata.olas@biol.uni.lodz.pl; Tel./Fax: +48-42-635-4485

**Keywords:** berry, lignans, phenolic compounds, safety, schisandrin, *Schisandra chinensis*

## Abstract

*Schisandra chinensis* (*S. chinensis*) berries, originally a component of traditional herbal medicine in China, Korea, and other east Asian countries, are also valuable agents in modern phototherapy. *S. chinensis* berry preparations, including extracts and their chemical components, demonstrate anti-cancer, hepatoprotective, anti-inflammatory, and antioxidant properties, among others. These valuable properties, and their therapeutic potential, are conditioned by the unique chemical composition of *S. chinensis* berries, particularly their lignan content. About 40 of these compounds, mainly dibenzocyclooctane type, were isolated from *S. chinensis*. The most important bioactive lignans are schisandrin (also denoted as schizandrin or schisandrol A), schisandrin B, schisantherin A, schisantherin B, schisanhenol, deoxyschisandrin, and gomisin A. The present work reviews newly-available literature concerning the cardioprotective potential of *S. chinensis* berries and their individual components. It places special emphasis on the cardioprotective properties of the selected lignans related to their antioxidant and anti-inflammatory characteristis.

## 1. Introduction

The genus *Schisandra* includes about 30 species. Among these, *Schisandra chinensis* (*S. chinenis*) Turcz. (Baill.), also commonly known as *Wuweizi* or *Beiwuweizi* in China, is a dioecious climbing plant. *S. chinenis* is a plant in the Magnoliaceae family. The meaning of *Beiwuweizi* is *Wuweizi* from the north of China. *Wuweizi* itself means fruit with five kinds of flavors: the sarcocarp and pericard are sweet and sour, the karmel is pepper and bitter, and the whole fruit tastes a little salty. In the past, it was believed that these five flavors affect the functioning of five organs: liver, lungs, kidneys, spleen, and heart. It is called gomishi in Japan, and omija in Korea [1,2,3,4].

*S. chinensis* is naturally found in north-eastern China (including Heilongjiang, Jilin, Liaoning, and Daxing’an Mountains), Japan, Korea, in the eastern part of Russia and in the south of the Sakhalin Island, and bears berries about 1 cm in diameter, whose exocarp and mesocarp are smooth, shiny, of a strong red color. Each berry contains one or two kidney-shaped seeds [1]. These fruits have a high potential for traditional herbal medicine for the treatment of different diseases, including cardiovascular diseases. Monograph of these berries is available in various Pharmacopoeias, including Chinese, Russian, Korean and Japanese, and are widely used in these countries. In the 2020 Edition of Chinese Pharmacopoeia, there were 100 prescriptions containing *S. chinenis* [4].

In food technology, *S. chinensis* berries are applied as additives to increase taste, flavor and nutritional value of food products. They are also believed to bestow beneficial health effects due to the presence of various bioactive compounds, including phenolic compounds, lipids, polysaccharides, and triterpenes [2,4]. These bioactive compounds are also found in seeds, shoots, and leaves. Various in vitro and in vivo studies have found them to demonstrate various antioxidative, anti-inflammatory, anticancer, and antiviral properties, among others. For example, *S. chinensis* has an inhibitory effect on cytochrome P450, family 3, subfamily A (CYP3A), and P-glycoprotein (P-gp), which can mediate metabolism or efflux of substrates, and may interact with many drugs [5,6,7,8,9,10,11,12]. In addition, Nasser et al. [9] described various biological properties of schisandrin B. A previous review by Chun et al. [13] indicated that *S. chinensis* berries demonstrate protective effects against common cardiovascular diseases (CVDs) such as hypertension and myocardial infarction. They described *S. chinensis* fruit extract and its lignans as promising resources for the development of safe, effective, and multi-targeted agents against cardiovascular diseases. The present paper examines this cardioprotective effect in more detail, as well as the effects of their components and associated food products, particularly those of selected lignans extracted from the berries. In addition, this review summarizes the cardioprotection properties of polysaccharides isolated from *S. chinensis*.

This review was performed following a search of papers identified in various electronic databases, including PubMed, Science Direct, Scopus, Web of Knowledge, Sci Finder, Web of Science, CNKI, Google Scholar, and the online ethnobotanical database. The last search was run on 20 December 2022. The following terms were used: “*S. chinensis*”, “*Schisandra chinensis*”, “fruits of *S. chinensis*”, “berries of *S. chinensis*”, “*Schisandra* lignans”, “*Schisandra* nortriterpenoids”, “*Schisandra* polysaccharides”, and “schisandrin”.

## 2. Phytochemical Characteristics of Various Parts of *S. chinensis*

**Figure 1:**
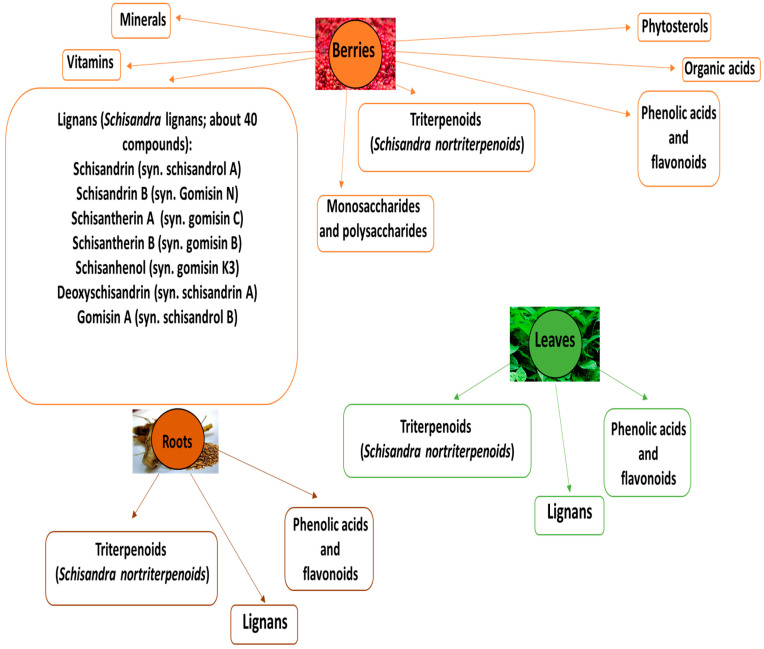
Bioactive compounds in various parts of *S. chinensis*.

Various bioactive compounds (about 300) have been isolated and identified in different parts of *S. chinensis*. Phytochemical studies have demonstrated that *S. chinensis* contains a large number of components, for example, lignans, phtosterols, essential oils, phenolic compounds, triterpenoids, and others, in which chemical structures of lignans and triterpenoids are unique, while other compounds. Bioactive compounds in various parts of *S. chinensis* are demonstrated in Figure 1. The precise phytochemical composition depends on a number of factors, including temperature, humidity, harvest time, light, geographical location, season, soil type, and maturity [1,14,15].

**Figure 2:**
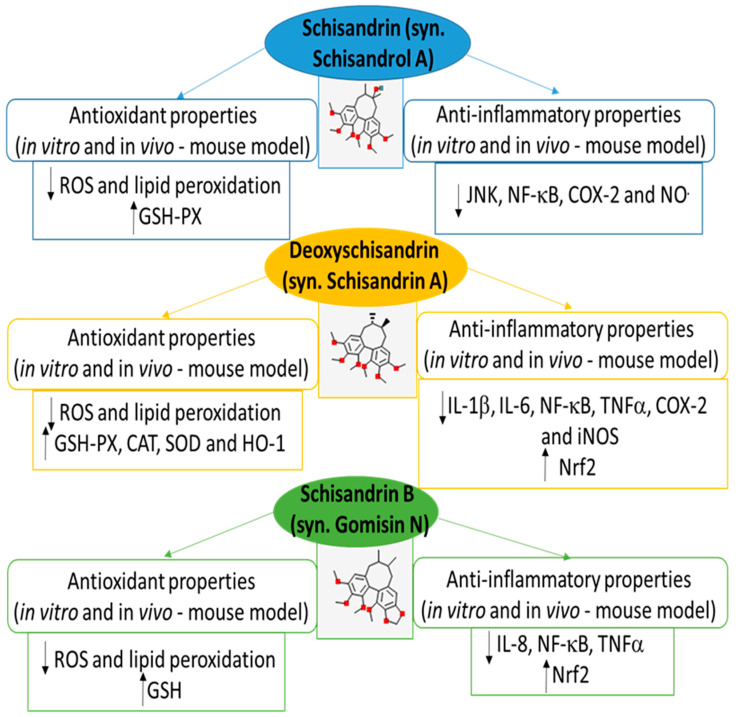
Biological activities and potential molecular mechanisms of cardioprotection by various selected lignans (schisandrin, deoxyschisandrin, and schisandrin B) from *S. chinensis* berries. ROS: reactive oxygen species; GSH-PX glutathione peroxidase; CAT: catalase; SOD: superoxide dismutase; HO-1: heme oxygenase-1; JNK: c-Jun *N*-terminal kinase; NF-κβ: nuclear factor kappa B; COX-2: cyclooxygenase-2; NO°: nitric oxide; IL-1β: interleukin-1β; IL-6: interleukons-6; TNFα: tumor necrosis factor-α; iNOS: inducible nitric oxide synthase; Nrf2: nuclear factor erythroid 2-related factor 2.

Lignans are the major bioactive components of the fruits of *S. chinensis* and possess various biological activities such as antioxidant, anticancer, anti-platelet, anti-inflammatory, and central nervous system protection, and lignans are mainly responsible for the pro-health properties of *S. chinensis*. Lignans are the class of secondary metabolites produced by oxidative dimerization of two phenylproponoid units. Lignans are one of the largest groups of naturally-occurring phenols in plants, and they are the major component of *S. chinensis* berries. In addition, these compounds can also be found in the shoots, leaves, and seeds. Moreover, lignans may be extracted from biomass of in vitro cultures. About 40 lignan types, mostly dibenzocyclooctanes, were isolated from *S. chinensis*. Among these, the most important active lignans are believed to be schisandrin, also denoted as schizandrin or schisandrol A or wuweizisu A by different authors, as well as schisandrin B (synonime (syn.) gomisin, wuwezisu B, γ-schisandrin), schisantherin A (syn. gomosin C, schisandrer A), schisantherin B (syn. gomosin B, schisanrer B), schisanhenol (syn. gomosin K3), deoxyschisandrin (syn. schisandrin A) and gomisin A (schisandrol B); each of these demonstrates unique biological activities. In the berries, the most abundant and active ingredient is schisandrin B [16]. More details about the chemical structure of *S. chinensis* lignans are described in another review paper [14]. Figure 2 demonstrates the chemical structures of selected lignans from *S. chinensis* berries.

The content of individual lignans in fruits varies considerably (4–19 g/100 g dry weight of the sample) and depends on the harvest season, the degree of fruit maturity, the location of the crop, and other ecological parameters [8,17,18,19]. For example, Liu et al. [19] reported that schisandrin constitutes 36–46% of all lignans in berries from China and 31–33% in those from Korea.

Importantly, *S. chinensis* lignans demonstrate a correlation between chemical structure and biological activity, including anticancer, antiviral, hepatoprotective, antioxidant, and anti-inflammatory properties. For example, a study of the protective action of three *S. chinensis* lignans (schisandrin A, B, and C) against myocardial ischemia-reperfusion injury found that the presence of a methylenedioxy group and the cyclooctadiene ring of schisandrin are significant determinants in the protection against myocardial ischemia-reperfusion injury [20]. In addition, Choi et al. [21] indicate that exocyclic methylene functionality plays a key role in the antioxidant properties of *S. chinensis* lignans.

The berries, leaves, seeds, and roots of *S. chinensis* are rich in phenolic acids (for berries: 9.20 ± 0.43 mg rutin equivalent (RE)/g plant material, for leaves: 62.36 ± 1.38 mg RE/g plant material) and flavonoids (for berries: 7.65 ± 0.95 mg RE/g plant material, for leaves: 35.10 ± 1.23 mg RE/g plant material) [22], and the berries, leaves, and roots also contain triterpenoids—lanostane (for example, kadsuric acid) and cycloartane-type triperpenoids (for example, schisanlactone D and wuweisilactone acid) and nortriterpenoids (for example, schindilactone A and wuweizidilactone I) often termed *Schisandra* nortriterpenoids or schinortriterpenoids. *S. chinensis* fruits are also sources of phytosterols (sitosterol, stigmasterol, and others), monosaccharides (glucose, fructose, arabinose, and galactose), polysacchraides, and various organic acids (citric, fumaric, tartaric and malic acids), minerals (for example Ca, B, Zn, Cr, Ni, Co, Mn, K. Cu, Fe, and Mg) and vitamins (C and E) [1,8]. For example, the analysis of monosaccharides composition demonstrated that *S. chinensis* polysaccharides contain 0.8% arabinose, 9.0% galactose, 64% glucose, and others. Their mass ranged from 18 to 127 kDa [23]. Results of Gao and Wu [24] demonstrated that conicasterol C is the most abundant component (22.02 ± 0.098 µg/mL) of *S. chinensis* oil.

A number of qualitative and quantitative analytical methods were developed due to the versatile bioactive activities of *S. chinensis* lignans. In addition to conventional extraction methods, supercritical carbon dioxide, ultrasounds, microwaves, and the application of supercritical antisolvent precipitation techniques were used to extract lignans from *S. chinensis* berries [16]. More details about the isolation, and chemical structure of *S. chinensis* lignans and other bioactive compounds are given in reviews by Szopa et al. [1], Li et al. [3], Sowndrararajan et al. [16] and Nowak et al. [8]. For example, Sowndrararajan et al. [16] described that lignans have protective action against different neuronal cell damage-mediated Parkinson’s disease, Alzheimer’s disease, stroke, and other neurodegenerative diseases. Moreover, these compounds enhance cognitive performance.

Few papers describe structure-biological activity relationships of lignans from *S. chinensis* [1,2]. For example about antioxidant properties of these compounds decided the exocyclic methylene group [1,2].

## 3. Cardioprotective Properties of *S. chinenis* Berries, their Components, and Food Products

*S. chinensis* berries and their extracts have been demonstrated to possess a range of biological properties, including cardioprotective ones, in vitro and in vivo. In addition, their components, especially lignans, may play an important role in the prophylaxis and treatment of cardiovascular diseases, including hypertension and myocardial infarction. These components, including schisandrin, are well-known Traditional Chinese Medicine formula ShengMai preparations, which act as a remedy for oxidative injury and can be used to treat various cardiovascular diseases [13,25,26,27].

In addition to oxidative stress, inflammatory processes also play important roles in the progress of cardiovascular diseases, including cardiomyopathy, atherosclerosis, and others. Demir [28,29] observed that paraoxanase-1 is an important protector enzyme against atherosclerosis; it protects low-density lipoprotein against oxidation. A key role in inflammation is played by vascular endothelial cells; for example, Hu et al. [30] have reported that the schisandra lignans demonstrate anti-inflammatory activity which inhibited the production of nitric oxide in lipopolysaccharide (LPS)—activated microglia cells (in vitro).

### 3.1. Cardioprotective Properties of S. chinensis Berry Preparations

In vitro and in vivo studies have found that preparations from *S. chinensis* berries exert their cardioprotective properties via various pathways including controlling inflammation, oxidative stress, and obesity [31,32,33,34,35]. Chen et al. [27] studied the effects of *S. chinensis* fructus berry ethanol extract on a rat model of atherosclerosis. The schisandrin, schisandrin A, and schisandrin B concentrations in the tested extract were found to be 291.8, 81.5, and 297.1 mg/g of dry weight, respectively, as determined by high-performance liquid chromatography (HPLC). *S. chinensis* extract (low-dose—0.35 g/kg/day, medium-dose—0.7 g/kg/day, and high-dose—1.4 g/kg/day) was administered through the intragastric route for three weeks. Aortic pathology changes, serum biochemical indices, and heme oxygenase-1 (HO-1) and nuclear factor erythroid 2-related factor 2 (Nrf-2) expression were measured. It was found that the tested extract lowers lipid peroxidation biomarkers, including malondialdehyde (MDA) level, and increases the activities of antioxidant enzymes, such as glutathione peroxidase (GSH-PX), catalase (CAT), and superoxide dismutase (SOD). *S. chinensis* extract also decreased the levels of thromboxane B_2_, endothelin-1, and oxidized-low-density lipoprotein (LDL). It is important to note that the three doses (0.35, 0.7, and 1.4 g/kg) used in this model were found to be free from toxic effects. On the basis of these results, authors suggest that the cardioprotective action of tested extract may be associated with increasing antioxidant capacity and improving endothelial dysfunction.

Park et al. [26] reported *S. chinensis* berry extract to have anti-obesity activity in induced obese rats. The tested extract was found to decrease body weight and fat tissue mass in high-fat diet obese rats.

It has known that anxiety and insomnia are often accompanied by cardiovascular dysfunction. However, a recent study by Su et al. [35] found that raw *S. chinensis* fruits, and the wine made from them, may alleviate cardiovascular dysfunction by promoting sleep in rats; they also suggest that promoting sleep by these products may increase the expression of melatonin receptor 1 (MT1) in the hypothalamus, and modulate the activity of hypothalamic-pituitary-adrenal (HPA) axis by regulating certain amino acid and neurotransmitter levels.

It Is worth noting that *S. chinensis* berries are available in pharmacies, herbal and online shops in dried form, as well as in the form of food products, such as teas, juices, tinctures or oils. Dried *S. chinensis* berry extract is also included in dietary supplements and can be purchased in capsule and tablet form, and as tinctures. Extracts are standardized for lignan content. It is generally recommended to take them in a dose of 500 mg to 2 g per day, after a meal. Extracts from *S. chinensis* berries can also be combined with other plant extracts, for example with *Panax ginseng* root extract, which has cardioprotective properties. One such dietary supplement, in tablet form, is Bodymax Vital (manufacturer: Axellus, Warsow, Poland); although its manufacturer claims it has a positive effect on the cardiovascular system, its exact cardioprotective potential is not described. According to the manufacturer’s state, the chemical composition of this supplement includes a standardized extract of *Panax ginseng* radix—50 mg, a standardized extract of *S. chinensis* berry—28 mg, and various vitamins and minerals. Another preparation, Full Spectrum Schizandra Berries 525 mg, in capsule form (manufacturer Swanson Health Products, New York, NY, USA) is recommended for normalizing blood pressure. In addition, other dietary supplements (Liveran, manufacturer HASCO-LEK, Warsow, Poland, in capsule form) helps to maintain normal blood lipid levels. Its chemical composition includes extract of *S. chinensis* berry—40–80 mg, extract of *Cynarae scolymus folium*—7–150 mg, extract of *Sylibum marianum* fruits—6.3–12.6 mg, silymarin—5–10 mg, vitamin B_1_—2 mg, vitamin B_2_—2 mg and choline—165 mg [2].

### 3.2. Cardioprotective Properties of S. chinensis Components

#### 3.2.1. Schisandrin (Schisandrol A)

Zhang et al. [36] examined the effect of schisandrin in vitro on a rat cardiomyocyte-derived cell line (H9c2) treated with LPS to model bacterial myocarditis. The cells were treated with two concentrations of schisandrin, 10 and 40 mM, for one to seven days. The authors observed that schizandrin promotes the recovery of myocardial tissues by enhancing cell viability and migration. Its mechanism of action includes downregulating SMAD family member 3 (Smad3), thereby reducing the activation of c-Jun *N*-terminal kinase (JNK) and nuclear factor kappa B (NF-κB) pathways [36].

Lai et al. [37] found that schisandrin treatment (6 mg/kg/day) relieved acute myocardial ischemia in ICR mice induced by coronary artery ligation. The compound improved various biochemical parameters and cardiac pathological alterations, preserved cardiac function, and decreased infarct size. In addition, it decreased the apoptotic index in rat cardiomyocyte cell line H9c2 in vitro when administered in two concentrations: 10 and 100 M. The findings suggest that the cardioprotective effect of schisandrin is based on the phosphoinositide 3-kinase/kinase Akt/NADPH oxidase 2 (PI3K/Akt-NOX2) signaling pathways.

Recently, Gong et al. [38] reported that schisandrin appears to attenuate myocardial ischemia/reperfusion-induced myocardial apoptosis through upregulation of 14-3-3*θ*. The authors also noted that the tested compound has antioxidant properties: it increased the activity of GSH-PX and reduced the level of MDA and reactive oxygen species (ROS). Schisandrin was found to demonstrate cardioprotective properties both in vivo in a myocardial ischemia/reperfusion injury mouse model (schisandrin A at 6, 12, and 24 mg/kg), and in vitro in a H9c2 cardiomyocyte cell line subjected to hypoxia/reoxygeneration injury (14, 28, and 56 µM).

#### 3.2.2. Schisandrin A

A number of experimental studies have demonstrated that schisandrin A has anti-inflammatory properties and that this may act by inhibiting proinflammatory signaling pathways or conversely, activating anti-inflammatory signaling pathways. Moreover, several in vitro and in vivo models have demonstrated that schisandrin A has antioxidant properties [4,15,39]. For example, Chang et al. [39] found schisandrin A to revieve myocardial ischemia-reperfusion injury in rats; it effectively protected cardiac function by reducing the left ventricular systolic pressure, left ventricular end-diastolic pressure, and occurrence of arrhythmia. It also reduced the level of oxidative stress, measured by the level of MDA, and SOD activity, as well as the apoptosis of myocardial cells and caspase-3 activity.

Zhou et al. [15] indicate that the compounds (80 mg/kg) alleviate cerebral ischemia-reperfusion injury by increasing SOD and CAT activity.

#### 3.2.3. Schisandrin B

Lin et al. [31] studied the anti-inflammatory potential of schisandrin B (10–50 µM) in LPS-stimulated human umbilical vein endothelial cells (HUVECs) in vitro. They reported that the tested compound suppresses the production of interleukin-8 (IL-8) and tumor necrosis factor-α (TNFα) following LPS treatment and that this suppression was blocked by transfection with Nrf2 siRNA. In addition, schisandrin B reduced LPS-stimulated intracellular adhesion molecule 1 (ICAM-1) and vascular cell adhesion molecule 1 (VCAM-1) expression. The authors suggest that the anti-inflammatory action of schisandrin B may include Nrf2 signaling pathway activation, which then inhibits LPS-induced inflammation in HUVECs.

Schisandrin B has been found to have a protective function against primary pulmonary hypertension by targeting transforming growth factor β1 [32], and attenuating angiotensin, II-induced endothelial-to-mesenchymal transition in the vascular endothelium by suppressing NF-κB activation [40]; the latter study used an in vitro model based on HUVECs treated with 10 µM schisandrin B, and an in vivo model where mice (*n* = 8) received orally schisandrin B (20 mg/kg, every day, for four weeks).

Interestingly, schisandrin B was also reported to avoid ischemia-reperfusion by exerting antioxidant activities [41]. Thandavarayan et al. [42] also observed that schisandrin B protects against oxidative stress in the heart in rodents, and that the compound may play an important role in the treatment of myocardial ischemia. It has observed that schisandrin B reduces myocardial injury through the inhibition of oxidative stress, and prevents apoptosis by decreasing the cleavage of caspase-3 and inducing Akt phosphorylation [43,44]. In addition, schisandrin B may increase mitochondrial glutathione production, which protects against ischemia/reperfusion injury by enhancing myocardial ATP [45,46].

Schisandrin B has also been found to reduce subcutaneous adipocyte size, subcutaneous adipose tissue mass and body weight in mice in vivo. In addition, treatment appears to reduce lipid concentration, and increase acid oxidation and lipolysis by the activation of protein kinase A (PKA) and hormone-sensitive lipase (HSL) in 3T3-L1 adipocytes in vitro [47].

#### 3.2.4. Schisandrin C

Han et al. [48] also discuss the potential value of schisandrin C as an antioxidant agent for treating vascular endothelial deficits. In an experiment where mice (*n* = 8) were treated with schisandrin C (10 mg/kg/day, for four weeks), they note that chisandrin C appears to target Kelch-like ECH-associated protein-1 (Keap-1), a negative regulator of Nrf2.

#### 3.2.5. Polysaccharides from *S. chinensis*

Twenty-four species of polysaccharides from *S. chinensis* were described. These polysaccharides have various molecular weights, monosaccharide composition and ratios, and structural characteristics, including the location of the functional groups. The generation of these differences may be due to different kinds of raw materials, production areas, purification processes, and parts of this plant (including berry, root, and stem), which may lead to the different biological functions of polysaccharides. Various studies demonstrated that these polysaccharides have different biological properties, including immunomodulatory, antioxidant, anti-cancer, anti-diabetic, anti-aging, analgesic, antitussive, anti-fatigue, hepatoprotective, hypolipidemic, and other. More details about the biological properties, functions, and application of these compounds are described in a review paper by Li et al. [3]. For example, Yue et al. [49,50] reported that polysaccharides from *S. chinensis* appear to possess antioxidant properties in vitro and in vivo. Interestingly, these antioxidant properties were significantly enhanced following selenylation.

A recent study by Shi et al. [23] found *S. chinensis* polysaccharide treatment (50 mg/kg) to prevent cardiac hypertrophy in C57/BL/6 mice (*n* = 6), possibly by dissociating thioredoxin-interacting protein/thioredoxin-1 complex and reducing oxidative stress. Two oxidative stress biomarkers were used: MDA concentration and SOD activity. Molecular docking analysis found that the tested polysaccharide binds to Arg207, Ser169, Lys166, Lys286, and Ser285 in thioredoxin-interacting protein (TXNIP), which is an endogenous inhibitor and regulator of thioredoxin-1 (Trx-1). Results of Shi et al. [23] suggest the potential use of these polysaccharides as a TXNIP inhibitor to attenuate oxidative stress, and TXNIP may represent a potential therapeutic target for the treatment of cardiac hypertrophy.

#### 3.2.6. Other Bioactive Compounds of *S. chinensis* Berries

Jang et al. [51] indicate that 10 and 30 µg/mL α-iso-cubebene (dibenzocyclooctadiene lignin found in *S. chinensis* fruits) appears to ameliorate the symptoms of cardiovascular diseases. This compound inhibits the proliferation of vascular smooth muscle cells by inhibiting the activator protein 1(AP-1) and CCAAT/enhancer-binding protein β (C/EBPβ) signaling pathways in vitro.

## 4. Conclusion

Various studies indicate that both *S. chinensis* berry preparations and their bioactive individual compounds, especially lignans and polysaccharides, appear to have cardioprotective potential; however, many of these works are limited to in vitro models. Nevertheless, the available literature indicates both to have anti-inflammatory, antioxidant, and anti-obesity potential [11,52]. For example, more details about the pharmacology and pharmacokinetics of bioactive compounds, including schisandrin A isolated from *S. chinensis* berries are described by Fu et al. [11] and Yang et al. [52]. In addition, various lignans from *S. chinensis* berries have been found to demonstrate cardioprotective potential and these are summarized in Figure 2, together with their mechanisms. For example, *S. chinensis* lignans demonstrate anti-inflammatory effects on different inflammatory models by regulating signaling pathways such as TNFα, NF-κB, Nrf2, NOS, and COX-2. The cardioprotective potential of *S. chinensis* berries seems to be associated with their antioxidative properties at the cellular level, as oxidative stress has been implicated in the pathogenesis of cardiovascular diseases, among others. The compounds have been found to effectively inhibit lipid peroxidation and ROS generation and to stimulate the activity of antioxidant enzymes, including SOD and CAT. In addition, the cardioprotective potential of various components of *S. chinensis* berries (in different in vitro and in vivo models) is described in Table 1.

One problem associated with research on phytocompounds or plant products involves standardization: it is difficult to compare results from different laboratories. In addition, the cardioprotective potential of *S. chinensis* lignans varies according to administration, model type, dosage, and experimental method. Moreover, cardioprotective potential has been only observed in vitro and in animal models, and there is no concrete clinical evidence for the efficacy, absorption, and bioavailability of *S. chinensis* lignans. Therefore, it is a hot topic in the future.

A number of dietary components, such as phenolic compounds and phytosterols may play a protective role in cardiovascular diseases. For example, the anti-platelet, anti-coagulant, anti-inflammatory, and antioxidant activities of phenolic compounds play a positive role in the prophylaxis and treatment of CVDs. In addition, phytosterols have also prophylaxis activities against hypercholesterolemia—which induces cardiovascular diseases. While it is significant that these compounds are present in *S. chinensis* berries, it is unknown whether they are more effective for the prophylaxis and treatment of cardiovascular diseases than lignans or *S. chinensis* berry products. Again, this matter needs further studies.

The safety of *S. chinensis* berry preparations has been reviewed in a number of articles [52,53]. Traditionally, *S. chinensis* is regarded as non-toxicity tonic. According to the 2020 Edition of Chinese Pharmacopeia, the recommended daily dose of *S. chinenis* is 2–6 g for an adult [52]. In addition, it has been demonstrated that *S. chinensis* extracts have little or no toxicity to pigs, rabbits, mice, and rats [52]. Moreover, Panossian and Wikman [53] have reported that ethanol extract of *S. chinensis* berries (even at very high doses) is non-toxic for experimental animals. Chen et al. [27] have also found the extract to be free from toxic effects in the model of atherosclerosis in rats. However, no concrete clinical experiments have investigated the human response to *S. chinensis* berry doses and their safety for treating different diseases, including cardiovascular diseases. In addition, there is a need for further studies on the interaction of *S. chinensis* berry preparations with various anticoagulant and antithrombotic drugs, such as clopidogrel, warfarin, apixaban, and aspirin. Recently, Valickova et al. [54] described that schisandrin and other compounds isolated from *S. chinensis* berries can negatively impact water biota in the case of massive contamination of surface water.

## Figures and Tables

**Table 1:** Cardioprotective potential of various components (selected lignans and polysaccharides) of *S. chinensis* berries.

Component of *S. chinensis* Berries	Biological Properties for Cardioprotective Action	References
Schisandrin (Schisandrol A, wuweizisu A)	Antioxidant properties (↓ROS and lipid peroxidation; in vitro model and in vivo (mouse) model) Anti-inflammatory properties (↓COX and NO°; in vitro model and in vivo (mouse) model)	[37,38,39]
Schisandrin A (deoxyschisandrin)	Antioxidant properties (↓lipid peroxidation and ↑CAT and SOD; in vitro model and in vivo model) Anti-inflammatory properties (in vitro model and in vivo model)	[4,15,39]
Schisandrin B (gomisin N, wuwezisu B, γ-schisandrin)	Anti-inflammatory properties (↓prostaglandins and NO°; in vivo model)	[43]
Schisandrin B (gomisin N, wuwezisu B, γ-schisandrin)	Anti-aggregation properties (in vitro model) Anti-inflammatory properties (in vitro model and in vivo model) Antioxidant properties (↓lipid peroxidation; in vitro model and in vivo model)	[31,40,41,42]
Schisandrin C	Antioxidant properties (↓lipid peroxidation; in vitro model and in vivo (mouse) model) Anti-inflammatory properties (↓COX and NO°; in vitro model and in vivo (mouse) model)	[48]
Polysaccharides	Antioxidant properties (in vitro model and in vivo model)	[23]

## Data Availability

Not applicable.

## Abbreviations

CAT, catalase; COX-2, cyclooxygenase-2; CYP3A, cytochrome P450, family 3, subfamily A; CVDs, cardiovascular diseases; GSH-PX, glutathione peroxidase; HO-1, heme oxygenase-1; HPA, hypothalamic-pituitary adrenal; HUVEC, human umbilical vein endothelial cell; ICAM-1, intracellular adhesion molecule 1; Il, interleukin; IL-1β, interleukin-1β; IL-6, interleukons-6; iNOS, inducible nitric oxide synthase; JNK, c-Jun *N*-terminal kinase; LDL, low-density lipoprotein; LPS, lipopolysaccharide; MDA, malondialdehyde; NF-κB, nuclear factor kappa B; NO°, nitric oxide; Nrf-2, nuclear factor erythroid 2-related factor 2; P-gp, P-glycoprotein; PI3K, phosphoinositide 3-kinase; RE, rutin equivalent; ROS, reactive oxygen species; SMAD3, SMAD family member 3; PKA, protein kinase A; *S. chinensis, Schisandra chinensis*; SOD, superoxide dismutase; TNFα, tumor necrosis factor-α; VCAM-1, vascular cell adhesion molecule 1.
